# Smart Homopolymer Microgels: Influence of the Monomer Structure on the Particle Properties

**DOI:** 10.3390/polym8040162

**Published:** 2016-04-23

**Authors:** Bastian Wedel, Yvonne Hertle, Oliver Wrede, Johannes Bookhold, Thomas Hellweg

**Affiliations:** Physical and Biophysical Chemistry, Bielefeld University, 33615 Bielefeld, Germany; bastian.wedel@tesa.com (B.W.); yvonne.hertle@uni-bielefeld.de (Y.H.); oliver.wrede@uni-bielefeld.de (O.W.); johannes.bookhold@uni-bielefeld.de (J.B.)

**Keywords:** NNPAM, NIPAM, NIPMAM, swelling behavior, microgel

## Abstract

In this work, we compare the properties of smart homopolymer microgels based on *N-n*-propylacrylamide (NNPAM), *N*-isopropylacrylamide (NIPAM) and *N*-isopropylmethacrylamide (NIPMAM) synthesized under identical conditions. The particles are studied with respect to size, morphology, and swelling behavior using scanning electron and scanning force microscopy. In addition, light scattering techniques and fluorescent probes are employed to follow the swelling/de-swelling of the particles. Significant differences are found and discussed. Poly(*N-n*-propylacrylamide) (PNNPAM) microgels stand out due to their very sharp volume phase transition, whereas Poly(*N*-isopropylmethacrylamide) (PNIPMAM) particles are found to exhibit a more homogeneous network structure compared to the other two systems.

## 1. Introduction

The formation of thermoresponsive microgels is a complex process, and it is of great interest to determine the structural and physical properties of these systems depending on the chemical structure of the used monomer and the synthesis conditions. A microgel basically consists of a cross-linker and a polymer which is responsible for the thermoresponsive properties of the gel network. If only one thermoresponsive polymer is present in the microgel, such systems are called homopolymers in the following.

Smart microgels are the subject of a steadily growing number of publications in recent years [1,2,3], which were reviewed by different groups focusing e.g., on core-shell microgels [4,5], copolymer microgels [6,7,8], soft nanotechnology [9,10], or on nanoparticle carriers [11,12,13,14,15]. For example, the particle size and the thermal behavior of *N*-isopropylacrylamide (NIPAM) [16,17] and *N*-isopropylmethacrylamide (NIPMAM) [18], depending on the initiator concentration, the reaction temperature and the cross-linker concentration, have been studied. However, to our knowledge, no systematic comparative study on monosubstituted acrylamides (NIPAM, NIPMAM, ...) exists, and the influence of their chemical structure on the particle formation and the thermoresponsive behavior of the resulting microgels. However, especially, these basic facts are very important to understand the formation of more complex systems like copolymer microgels or core-shell particles, which are suitable for a lot of different applications due to their smart behavior [19,20]. For this purpose, the crucial point is a precise knowledge about the controllability of the microgel properties. Particularly, the particle size, the volume phase transition temperature (VPTT) as well as the shape of the phase transition curve is very important. The easiest way to understand the interplay between microgel properties, monomer structure and synthesis conditions is to study homopolymers at first.

In this contribution, we want to focus on homopolymer microgels based on NIPAM, NIPMAM and *N-n*-propylacrylamide (NNPAM). The influence of the monomer chemical structure on the microgel particle size, the morphology and the swelling behavior is studied and compared in detail. Generally, all synthesis have been performed as precipitation polymerisation without surfactant and under identical conditions to ensure a good comparability of the results. Besides imaging techniques and light scattering, the study of fluorescence probes inside the microgel is used to scrutinize the volume phase transition of the different microgels.

## 2. Materials and Methods

### 2.1. Chemicals

*N*-isopropylacrylamide (NIPAM; Sigma-Aldrich Chemie GmbH Munich, Germany; purity 97%) and *N*-isopropylmethacrylamide (NIPMAM; Sigma-Aldrich, Chemie GmbH, Munich, Germany; purity 97%) were purified by recrystallisation from hexane. The cross-linker *N,N*’-methylenebisacrylamide (BIS; Sigma-Aldrich Chemie GmbH, Munich, Germany; purity 99%), the initiator ammonium persulfate (APS; Sigma-Aldrich Chemie GmbH, Munich, Germany; purity ≥ 98%) and pyrene (Sigma-Aldrich Chemie GmbH, Munich, Germany; purity ≥ 99%) were used without further purification. For all experiments, purified water from an Arium®pro VF system (Sartorius AG, Göttingen, Germany) was used.

*N-n*-propylacrylamide (NNPAM) was synthesized via a Schotten–Baumann reaction published by Hirano *et al.* [21]. For this reaction, acryloylchloride (Sigma-Aldrich Chemie GmbH, Munich, Germany; purity 98%), *n*-propylamine (Fluka, Sigma-Aldrich Chemie GmbH, Munich, Germany; purity 99%), triethylamine (Grüssing GmbH Analytika, Filsum, Germany; purity 99%) and methylenchloride (p.a.) were used as received. The obtained monomer NNPAM was washed with NaHCO${}_{3}$ (10 wt %) and dried over MgSO${}_{4}$. After filtration, the solvent was evaporated and the product was distilled in vacuum (115 ${}^{{}^{\circ}}$C, 10 mbar).

### 2.2. Synthesis of the Homopolymer Microgels

The homopolymer microgels of NNPAM, NIPAM and NIPMAM were synthesized via conventional precipitation polymerization without surfactant. All synthesis were performed in a 250 mL three-neck flask equipped with a reflux condenser, mechanical stirrer and a nitrogen inlet. The monomer (11.05 mmol) and the cross-linker *N,N*’-methylenebisacrylamide (BIS) (5.4 mol % respective to the total monomer amount) were dissolved in 150 mL purified water and were heated up to 70 ${}^{{}^{\circ}}$C under continuous stirring and purged with nitrogen. After 1 h the polymerization was initiated by the addition of 1 mL of a 2.71 mM solution of APS and left to proceed for 4 h at 70 ${}^{{}^{\circ}}$C. After the reaction time, the solution was cooled to room temperature and stirred over night. For purification, all samples have been treated by five cycles of centrifugation, decantation and redispersion using purified water.

### 2.3. Scanning Electron Microscopy

SEM investigations were performed on a ESEM-FEG (Philips XL30, Eindhoven, Netherlands) with an acceleration voltage of 3 kV and working distances between 5 and 6 mm. For the sample preparation 50 μL of microgel solution ($c∼5\times 10^{-4}\;\mathrm{wt}\%$) were deposited on cleaned silicon wafers and dried at room temperature in air. The completely dried samples were sputtered (Bio-Rad Laboratories GmbH, Munich, Germany, model E5000) with a thin layer of gold (≈ 3 nm) to increase the conductivity and the contrast in SEM. Before use, the Si wafers have been cleaned with ethanol and afterwards treated with oxygen plasma for 10 min. Then, for high purity, the wafers were additionally cleaned with a solution containing H${}_{2}$O:NH${}_{3}$:H${}_{2}$O${}_{2}$ in a ratio of 5:1:1 [22]. The particle diameters were analyzed using the program ImageJ (Wayne Rasband, National Institutes of Health, USA) [23].

### 2.4. Atomic Force Microscopy

The Atomic Force Microscopy (AFM) images were recorded using a Nanoscope III (Digital Instruments, now Brucker, Karlsruhe, Germany) working in tapping mode. The samples were prepared on cleaned silicon wafer which have been cleaned before as described in the cleaning procedure in Section 2.3. Fifty microlitres of microgel solution with a concentration of $c∼5\times 10^{-4}\;\mathrm{wt}\%$) was deposited on the substrate and dried at room temperature in air. The used cantilevers were from Budget Sensors (Innovative Solutions Bulgaria Ltd., Sofia, Bulgaria) (Tap300Al-G) with a radius of ≤10 nm, a resonance frequency of about 300 kHz and a spring constant of 40 N/m.

### 2.5. Light Scattering

#### 2.5.1. Photon Correlation Spectroscopy

For the dynamic light scattering experiments, two different setups were used. The angle dependent measurements were done using an ALV goniometer setup equipped with a multiple-*τ* digital correlator ALV-5000/E (ALV-Laser Vertriebsgesellschaft mbH, Langen, Germany) and an argon-ion laser (Spectra Physics Stabilite 2017, Newport Spectra-Physics GmbH, Darmstadt, Germany; *λ* = 514.5 nm) as light source. The temperature dependent measurements were performed at a constant scattering angle of 60 ${}^{{}^{\circ}}$ with a solid-state laser at *λ* = 661.4 nm (TOPTICA Photonics AG, Graefelfing, Germany). To detect the time-intensity-autocorrelation function in this second setup, an ALV-6010 multiple-*τ* correlator (ALV-GmbH, Langen, Germany) was employed. In every case, the sample temperature was adjusted using a temperature controlled decaline matching bath and all measurements were repeated at least three times. The concentration of the microgel solutions was below 0.001 wt % to avoid multiple scattering. The analysis of the measured autocorrelation functions was done by inverse Laplace transformation [24,25] or with the method of cumulants [26,27] and provides the mean relaxation rate $\bar{\Gamma }$ of the relaxation rate distribution function. From this, the translational diffusion coefficient $D^{T}$ for diluted particle solution can be calculated via $\bar{\Gamma }=D^{T}\cdot q^{2}$ with *q* as magnitude of the scattering vector (q=4πn/λ·sin(θ/2), with *λ* being the wavelength of the used radiation, *n* the refractive index and *θ* the scattering angle). Furthermore, using the Stokes–Einstein relation, a calculation of the hydrodynamic radius $R_{h}$ of the microgel particles is possible:

(1) $$D^{T}=\frac{k_{B}T}{6\pi \eta R_{h}},$$

$k_{B}$ is the Boltzmann constant, *T* the temperature during the measurement and *η* the corresponding viscosity of the solvent. For a correct evaluation of the obtained data, the values for the refractive index and the viscosity of water were corrected with respect to the measurement temperature [28].

#### 2.5.2. Static Light Scattering

The static light scattering experiments were done applying an ALV/CGS-3 compact goniometer system with a helium-neon laser (JDSU 1145/P, JDS Uniphase Corporation, now Viavi Solutions INC ,Milpitas, USA; *λ* = 632.8 nm) and toluene as matching bath. The detected intensity of the scattered light for the sample, the solvent, and the standard at different angles was corrected using the following equation (index *i*: sol = solvent, s = sample or sta = standard):

(2) $$I_{i,\Theta }=\frac{CR_{i,\Theta }\cdot \sin\left(\Theta \right)}{I_{\text{laser}}}.$$

Here, $I_{i,\Theta }$ is the detected intensity at a scattering angle Θ, $I_{\text{laser}}$ is the incident laser intensity (measured via an additional monitor diode) and $CR_{i,\Theta }$ the measured count rate at the detector. To scale all measured scattering intensities to absolute values $I_{\text{abs},\Theta }$, Equation (3) is used, where $I_{s,\Theta }$, $I_{\text{sol},\Theta }$, and $I_{\text{sta},\Theta }$ are the corrected values from Equation (2). $R_{\text{sta},\Theta }$ is the Rayleigh ratio of the standard, $n_{\text{sol}}$, and $n_{\text{sta}}$ are the refractive indices.

(3) $$I_{\text{abs},\Theta }=\frac{I_{s,\Theta }-I_{\text{sol},\Theta }}{I_{\text{sta},\Theta }}\cdot R_{\text{sta},\Theta }\cdot {\left(\frac{n_{\text{sol}}}{n_{\text{sta}}}\right)}^{2}.$$

Additionally, the intensity $I_{\text{abs},\Theta }$ was corrected with respect to the back reflection of the laser beam at the glas/solvent interface. Therefore, the intensity $I_{180-\Theta }$ at a scattering angle of 180 ${}^{{}^{\circ}}$ and the reflection coefficient *r* of solvent/borosilicate is used:

(4) $$I_{\text{refl}.\text{corr},\Theta }=I_{\text{abs},\Theta }\cdot \left(\frac{1-2\cdot r\cdot \frac{I_{180-\Theta }}{I_{\text{abs},\Theta }}}{{\left(1-r\right)}^{2}}\right).$$

### 2.6. Turbidity Measurements

The light attenuation in microgel solutions is generally caused by the scattering of light by the dispersed particles. For large particles (size of the order of the wavelength of light), and when the particle interior is optically very different from the surrounding medium, the Rayleigh–Debye–Gans (RDG) approximation is no longer valid [29]. A criterion to judge the validity of RDG approximation is given by:

(5) $$\frac{4\pi }{\lambda }R\left|m-1\right|\ll 1.$$

Here, *R* is the radius of the particle, *m* is the ratio between the refractive index inside and outside the particle and *λ* the wavelength of the used radiation. In the case of our homopolymer microgels, the effective refractive index at low temperatures (swollen particles) is sufficiently low, and, therefore, the Rayleigh–Debye–Gans approximation holds in this present case [30]. In contrast to this, at high temperatures (collapsed particles), the refractive index of the microgel is different compared to that of water (n= 1.46 [30]) and the experimental data would have to be described by a Mie calculation for a homogeneous sphere. This is relevant in the data treatment of SLS, but not for the turbidity measurements where only the intensity of the transmitted light $I_{T}$ at an angle of 180 ${}^{{}^{\circ}}$ is analyzed. However, in the present work, we only studied the swollen particles by static light scattering (SLS) and the RDG approximation holds.

Hence, in the turbidity measurement, the scattering from the particles is a combination of differences in the refractive index (between particles and solvent) and of the particle size. Summarizing all processes, the incident light intensity $I_{0}$ is reduced by scattering (S) and by absorption (A) and for the measured transmitted intensity $I_{T}$ the following equation holds:

(6) $$I_{T}=I_{0}-I_{S}-I_{A}.$$

For microgels, the absorption $I_{A}$ is negligible and the decrease of the transmitted intensity is nearly exclusively caused by scattering processes. For a quantitative description of the intensity of the transmitted light, the Lambert–Beer law at low sample concentrations can be used. $D_{\lambda }$ is the light attenuation, *c* the sample concentration, *d* the thickness of the measurement cell and $\delta_{\lambda }$ the attenuation coefficient:

(7) $$D_{\lambda }=\mathrm{lg}\left(\frac{I_{0}}{I_{T}}\right)=\delta_{\lambda }\cdot c\cdot d.$$

During the volume phase transition of microgel particles, the light attenuation $D_{\lambda }$ changes due to a reduction in particle size and a simultaneous increase of the refractive index. This characteristic behavior can be followed by UV/Vis spectroscopy [31,32,33,34]. Therefore, light of one wavelength is focused on the sample and the intensity of the transmitted light at an angle of 180 ${}^{{}^{\circ}}$ at different temperatures is detected. For a better comparison of the phase transition of different microgel solutions the light attenuation $D_{\lambda }$ is normalized to the sample concentration (in wt %) and the thickness of the cuvette (1 cm), and the normalized attenuation coefficient $\delta_{\lambda }$ is given by:

(8) $$\delta_{\lambda }=\frac{D_{\lambda }}{c\cdot d}.$$

For the turbidity measurements, a UV-Visible spectroscopy system (Agilent 8453, Agilent Technologies Deutschland GmbH, Ratingen, Germany) with a sample changer with 8 positions was used and the attenuation at a wavelength of 700 nm was analyzed. To measure the temperature dependent swelling behavior of the microgels, a constant heating rate of 3 ${}^{{}^{\circ}}$C/h was chosen and the turbidity was detected every 30 s. For a good signal to noise ratio, the concentration of the samples was set between 0.0015 wt % and 0.25 wt %.

### 2.7. Fluorescence Measurements

The fluorescence experiments were performed with a FP-8300 spectrometer from Jasco (Jasco Labor- u. Datentechnik GmbH, Groß-Umstadt, Germany) equipped with a Peltier temperature controlled sample holder for 4 samples. The temperature equilibration time was 10 min for each sample and the excitation/emission bandwidth was 2.5 nm. The excitation of pyrene was carried out at 336 nm and the fluorescence signal was detected in a range from 350 up to 500 nm with an accuracy of 0.5 nm. For the measurements microgel solutions with concentrations between 0.01 wt % and 0.1 wt % were mixed with a saturated pyrene solution ($c_{\text{Pyrene}}\approx$ 6 μM) and measured in 10 mm quartz Hellma cells.

## 3. Results and Discussion

### 3.1. Imaging Techniques

**Figure 1:**
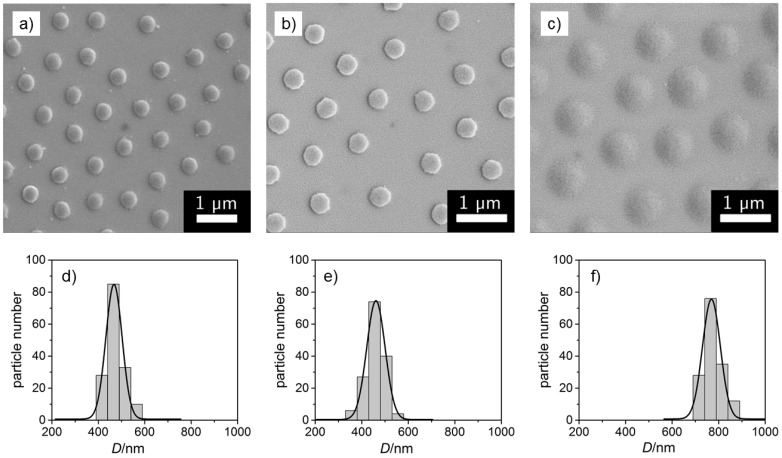
SEM images of the homopolymer microgels of (**a**) NNPAM, (**b**) NIPAM and (**c**) NIPMAM synthesized without surfactant and the corresponding size distributions (**d**–**f**). The black lines represent a Gaussian distribution function.

The three different homopolymer microgels were deposited on Si-wafers, sputtered with a thin layer of gold and imaged by scanning electron microscopy (SEM) as represented in Figure 1. The diameters of the particles were graphically analyzed leading to the size distributions displayed below the SEM images. It is clearly observable that the polydispersities of the microgel particles are very low in all cases. Table 1 gives the averaged particle diameters ${\bar{D}}_{\text{SEM}}$ and the respective standard deviation obtained from the half width at half height of the distributions shown in Figure 1.

**Figure 2:**
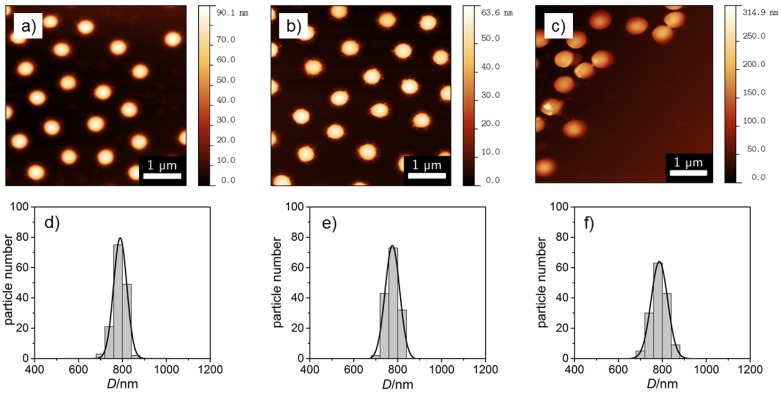
AFM images of the homopolymers of (**a**) NNPAM, (**b**) NIPAM and (**c**) NIPMAM synthesized without surfactant and the corresponding size distributions (**d**–**f**). The black lines represent a Gaussian distribution function.

The corresponding height images of the three homopolymer particles observed by atomic force microscopy (AFM) using tapping mode are represented in Figure 2, and a summary of the obtained diameters ${\bar{D}}_{\text{AFM}}$ are also given in Table 1. From the pictures, it is clear that both methods prove the low polydispersity of the particles. However, a direct comparison of the measured particle radii reveals differences in size for NNPAM and NIPAM depending on the imaging techniques which is used, while, for NIPMAM, the results match within the experimental error (see Table 1).

**Figure 3:**
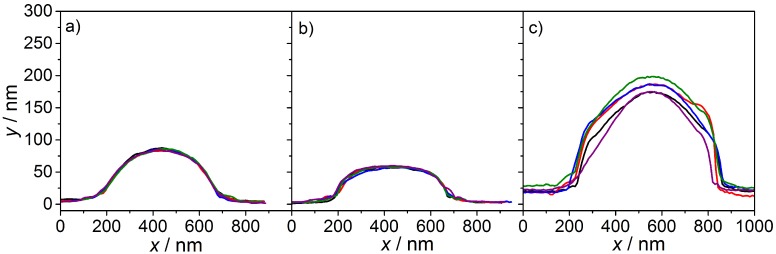
From left to right: height profiles of (**a**) NNPAM, (**b**) NIPAM and (**c**) NIPMAM homopolymer microgels obtained from AFM measurements in tapping mode. Each line represents one microgel particle of the same sample. The samples are characterized in the dried state.

The effect of the size differences can be caused by the network structure of the microgel particles in combination with substrate interactions and the way the images are taken. In short, it is known that most acrylamide microgels exhibit an inhomogeneous distribution of the cross-linker due to a different polymerization rate of BIS in contrast to the acrylamide monomers. Therefore, a BIS-gradient from a more dense core to a less cross-linked shell is observed [34,35,36,37]. If these microgels are now deposited on a Si-wafer as substrate, the soft particles change their size due to the drying process required for AFM and SEM. This results in a stiff core area (similar to a hard sphere) and a thin, flat and less cross-linked outer shell. Depending on the characteristics of the cross-linker gradient and on the imaging technique it is not possible to resolve all parts of the microgel particle. With SEM the thin fuzzy shell could not be monitored (but the core) and the particles appear to be smaller than in reality. In contrast to this, with AFM, it is possible to image very thin samples. Hence, it can be assumed that NIPAM and NNPAM consist of a more dense core and a less cross-linked shell while NIPMAM form more homogeneous microgel particles. A more detailed analysis of the AFM height images of all three homopolymer particles shows that, for all microgels, the diameter is nearly the same, while for PNIPMAM, a larger vertical dimension is measurable (see Figure 3). This can be caused by different effects like interactions with the substrate, the stiffness of the microgels or even the molecular weight of the particle. However, unfortunately at this point, the method does not clarify if the particles comprise a core-shell structure or not.

**Figure 4:**
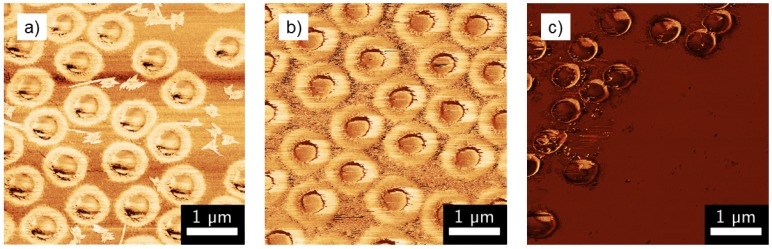
Phase images of (**a**) PNNPAM, (**b**) PNIPAM and (**c**) PNIPMAM homopolymer microgels obtained from AFM measurements.

From the AFM measurements, it is also possible to obtain the phase images which contain information on the rigidity of the material of observation [38]. Therefore, a core-shell particle with different degrees of rigidity of the core and the shell should be clearly identifiable. These phase images of the three homopolymer microgels are presented in Figure 4.

These images reveal the presence of a defined core-shell structure in the case of the PNNPAM and PNIPAM microgels (with a higher rigidity in the inner area of the particles), whereas for PNIPMAM, a more homogeneous structure is observed. A calculation of the inner diameter on the basis of the AFM phase images yields a value of 483 nm for PNNPAM and 461 nm for PNIPAM, which is in good agreement with the results from the SEM measurements (see Table 1). Analyzing the phase image of PNIPMAM results in a nearly uniform rigidity for the whole particles, and, as a consequence, these particles seem to consist of a weakly but rather homogeneously cross-linked network. However, it has to be mentioned here that the imaging techniques AFM and SEM provide only a rough indication of the structure of the particles and that the microgels are only characterized with these methods in the dried state. Therefore, additional techniques to study the homopolymer particles in bulk solution have to be used.

### 3.2. Static Light Scattering (SLS)

**Figure 5:**
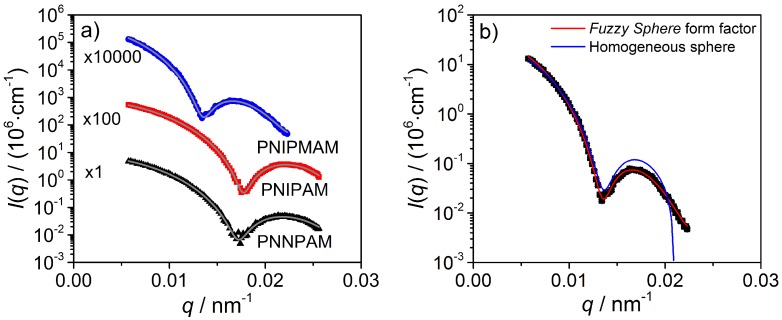
Plot of the absolute scattering intensities vs. the scattering vector *q* of PNNPAM, PNIPAM and PNIPMAM at 15 ${}^{{}^{\circ}}$C and the corresponding fit with the fuzzy sphere model (**left**) [17]. For a better representation of the experimental data, the intensity values were shifted along the *y*-axis. Comparison of the homogeneous [39] and the fuzzy sphere form factor model on the example of PNIPMAM (**right**).

To obtain information about the microgel solution structure, static light scattering experiments at a temperature of 15 ${}^{{}^{\circ}}$C and in an angular range from $15{\;}^{{}^{\circ}}$ to $155{\;}^{{}^{\circ}}$ were performed. The low measurement temperature was chosen to ensure that all polymer particles are in the totally swollen state since the lower critical solution temperature (LCST) of PNNPAM is about 21 ${}^{{}^{\circ}}$C. In Figure 5a, the absolute scattering intensities as a function of the scattering vector *q* and the corresponding fits are illustrated.

A quantitative analysis of the scattering curves was done by using a homogeneous sphere form factor [39] as well as a fuzzy sphere form factor (Equation (9)) [17]. The homogeneous sphere model does not properly describe the intensity of the first resolved form factor maximum, whereas the latter model is in very good agreement with the experimental data (see Figure 5a). As an example for the differences in the two form factor models, the scattering curves of PNIPMAM with the fits obtained by the homogeneous and the fuzzy sphere model are shown in Figure 5b. To take the size polydispersity of the particles into account, a Gaussian distribution function for the radius *R* is assumed [17]:

(9) $$\left[H\right]P{\left(q\right)}_{\text{fuzzy}\;\text{sphere}}={\left[\frac{3(\sin(qR)-qR\cos(qR)}{{\left(qR\right)}^{3}}\cdot \exp\left(-\frac{{\left(\sigma q\right)}^{2}}{2}\right)\right]}^{2}$$

The fuzzy sphere model describes a microgel particle with two different zones. The polymer density distribution of an inner area (core) can be described by means of a radial boxprofile with the radius $R_{\text{box}}=R-2\sigma$. The outer zone (shell) is described by an exponential density gradient. At the radius *R*, the polymer density of the core is reduced to one half and *σ* takes the cross-linking gradient into account. From the static light scattering experiment in combination with the fuzzy sphere model, the radius of the whole particle with $R_{\text{particle}}=R+2\sigma =R_{\text{box}}+4\sigma$ is available, and, accordingly, the *σ*-value in this equation provides information on the inhomogeneity of cross-linking (low *σ*: homogeneous sphere; high *σ*: strong gradient).

From the experimental data and the obtained values from the fits (see Table 2), it is clear that all microgel particles exhibit an inhomogeneous density distribution which is in agreement with other results from literature for PNIPAM [17,40] and PNIPMAM [41]. The form factor minimum at $q_{\text{min}}$ for PNNPAM and PNIPAM is nearly at the same position (*q* = 1.73 $\times 10^{-2}$ nm${}^{-2}$/1.77 $\times 10^{-2}$ nm${}^{-2}$), which indicates that the particles have the same size. The $q_{\text{min}}$ value for PNIPMAM is shifted to smaller *q*-values (1.35 $\times 10^{-2}$ nm${}^{-2}$), and, therefore, the particle size is increased compared to the others. The other obtained parameters from the fuzzy sphere model for the radius of the homopolymer microgels, the polydispersity and the *σ*-value, are summarized in Table 2.

**Figure 6:**
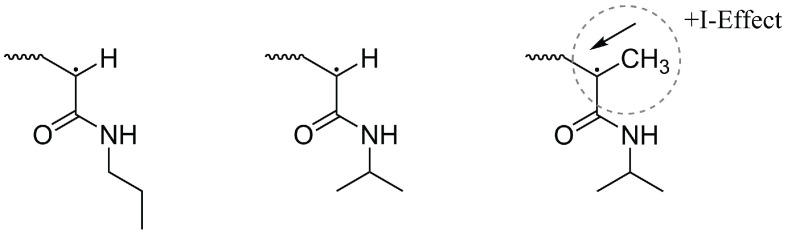
Structural formulas of the various acrylamide radicals during polymerisation (left to right: NNPAM, NIPAM and NIPMAM). In NIPMAM, the radical is stabilized by the methyl group and its positive inductive effect, whereby the polymerization rate is reduced.

Notably, the homopolymer particles differ in size, although they have been prepared under identical conditions. The obtained diameter for PNNPAM and PNIPAM is nearly the same (300 nm/284 nm), while PNIPMAM deviates (464 nm). This can be explained by the differences in the molecular structure of the monomers and hence with their polymerization rate (connected to the amount of precursor particles build in the first step of the emulsion polymerization). NIPMAM has an additional methyl group at the growing polymer radical and due to hyperconjugation a positive inductive effect (+I - effect) causes a stabilization of the radical end (see Figure 6). Therefore, the growing polymer radical of NIPMAM is more stable in contrast to the radical of NIPAM and NNPAM. Consequently, a lower amount of precursor particles is formed at the beginning of the polymerization and the resulting particles are larger at the end.

The *σ* value obtained from the fuzzy sphere analysis of the SLS measurements (see Table 2 last column) yields an indication about the density gradient inside the different homopolymer microgels. From this, a comparison of the whole particle radius $R_{\text{particle}}$ in contrast to the compact core radius from the boxprofile $R_{\text{box}}$ is possible. In the case of PNNPAM and PNIPAM the core engross nearly 75% of the particle while for PNIPMAM it’s only about 42%. Therefore, three quarters of the PNNPAM and PNIPAM particles are densely cross-linked in the core region and exhibit a strong cross-linker gradient in a small outer shell. This core-shell structure matches with the results from the AFM measurements under the limitation in AFM the microgels are in the totally collapsed state. For the microgel based on PNIPMAM, there exists a smaller core area and the shell with a continuous, rather flat cross-linker gradient, is more pronounced. This is in line with the AFM phase images which show no rapid change in rigidity for these microgel particles.

But why are these particles so differently cross-linked? The reason for this is the difference in the polymerization rates for the cross-linker BIS in combination with the different acrylamide monomers. In the case of NIPMAM, the cross-linker is mainly consumed at the beginning of the polymerization and builds oligomers without a temperature sensitivity. Afterwards, due to the low polymerization rate of NIPMAM, oligomers (and later particles) of this monomer with a low amount of BIS are formed, leading to lowly cross-linked microgel particles. The pre-build BIS-oligomers might later absorb to the growing PNIPMAM microgel due to hydrophobic interactions and maybe incorporated in the microgel network. The disadvantage of this oligomer/polymer building sequence is that a high amount of water-soluble polymer, which is not incorporated in the microgel particles, is generated. This assumption was confirmed by analyzing the amount of residual water-soluble polymer after the purification (centrifugation) of the microgel particles. Using NIPMAM/BIS as monomers, nearly 50% of the generated polymer is not part of the obtained microgel particles. For NNPAM and NIPAM, the amount of side-product is between 10% and 19%. Thus, during the formation of the NIPMAM particles compared to NNPAM and NIPAM, a significantly smaller amount of the monomer is used and the cross-linking density deviates strongly between the different homopolymer microgels.

### 3.3. Swelling Behavior

#### 3.3.1. Photon Correlation Spectroscopy (PCS)

**Figure 7:**
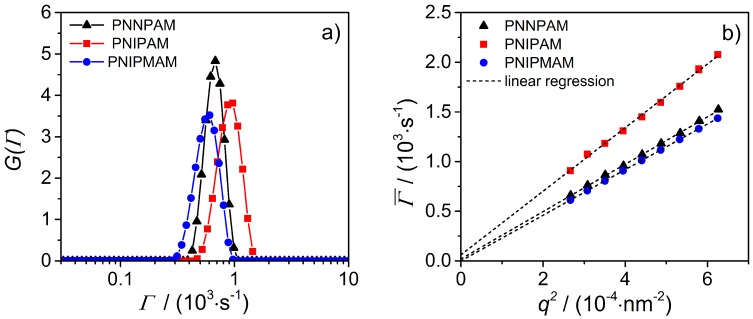
(**a**) PCS relaxation rate distribution of the collapsed homopolymer microgels; (**b**) Plot of the mean relaxation rate $\bar{\Gamma }$ *vs.* $q^{2}$ and the corresponding linear regression to determine the translational diffusion coefficient $D^{T}$.

In a typical experiment, the particle motion in solution is analyzed and from this the particle size can be calculated [29] if only translational diffusion is observed. To ensure that this is the case, angle dependent PCS measurements of the microgels in the swollen and collapsed state have been performed (PNNPAM: 15 ${}^{{}^{\circ}}$C and 40 ${}^{{}^{\circ}}$C; PNIPAM: 20 ${}^{{}^{\circ}}$C and 50 ${}^{{}^{\circ}}$C; PNIPMAM: 30 ${}^{{}^{\circ}}$C and 60 ${}^{{}^{\circ}}$C) and the obtained mean relaxation rates $\bar{\Gamma }$ were plotted against $q^{2}$. The analysis of the autocorrelation function was done by inverse Laplace transformation (CONTIN) and also by the cumulant method. The results obtained from both methods were the same. Hence, only the values from CONTIN are presented in the following. As an example, the relaxation rate distribution of the collapsed homopolymer microgels at a *q*-value of 1.63 $\times 10^{-2}$ nm${}^{-1}$ is illustrated in Figure 7a.

It is clearly visible that all microgel samples show a monomodal relaxation rate distribution with a narrow full width at half maximum. Both facts confirm the presence of only one particle species with a low polydispersity. Therefore, the results are in good agreement with those from the imaging techniques. An analogous behavior was found for all particles in the swollen and collapsed state at all scattering angles. Plotting the relaxation rate as a function of the magnitude of the scattering vector $q^{2}$ results in a linear dependence which can be fitted by a line through the origin (see Figure 7b). Hence, the monitored dynamic of the system is only translational diffusion of the particles and from the slope of the linear fit the translational diffusion coefficient can be calculated. Using the Stokes–Einstein equation (see Equation (1)), the hydrodynamic radius of the microgels was determined and the results are summarized in Table 3. $R_{h}$ for the swollen and collapsed PNNPAM and PNIPAM microgels, respectively, are identical within the experimental precision. In contrast, the value for PNIPMAM particles differs clearly. This is in good agreement with the results from the SLS experiments.

From the particle size, the maximum swelling ratio $\alpha_{\text{max}}$ according to Equation (10) can be calculated. In Equation (10), $V_{\text{swollen}}$ and $V_{\text{collapsed}}$ represent the particle volume in the swollen and collapsed state, respectively. Based on the assumption that the microgel particles are spherical, the swelling ratio $\alpha_{\text{max}}$ is given by:

(10) $$\alpha_{\text{max}}=\frac{V_{\text{swollen}}}{V_{\text{collapsed}}}=\frac{R_{h,\text{swollen}}^{3}}{R_{h,\text{collapsed}}^{3}}.$$

**Figure 8:**
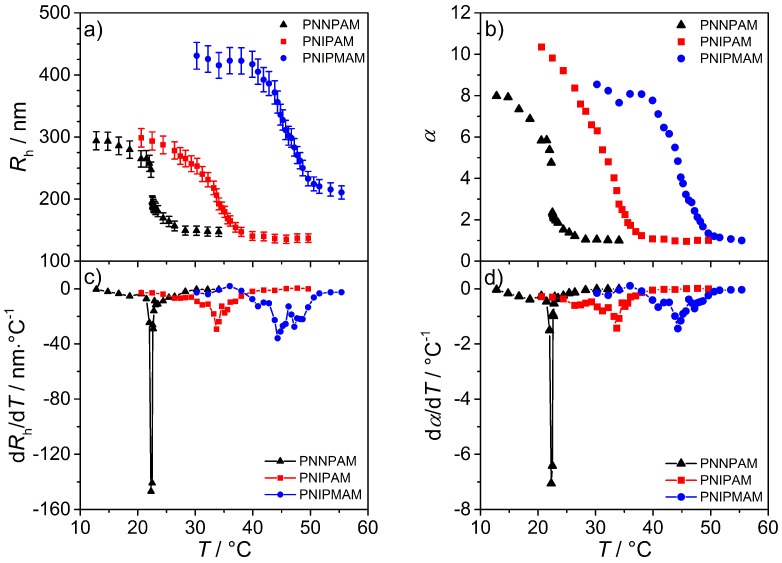
The swelling curves show the change of the microgel hydrodynamic radius (**a**) and the swelling ratio *α* (**b**) as a function of temperature for all three homopolymers. The graphs (**c**) and (**d**) represent the numerically calculated first derivative of the swelling curves. The point of inflection is the volume phase transition temperature (VPTT) of the system.

To characterize the thermoresponsive behavior of the homopolymer microgels and to determine the VPTT, temperature dependent measurement of the hydrodynamic radius were additionally performed. The respective swelling curves of the homopolymer particles are illustrated in Figure 8a. In general, with increasing temperature the solubility of the acrylamide polymer network decreases and as a consequence the network collapses and the particle size is reduced. To determine the VPTT, an analysis of the point of inflection of the swelling curves was performed. For this purpose, the curves were first numerically differentiated (Figure 8c) and then the maximum of the derivative was determined. The obtained values for the VPTT are 22.3 ${}^{{}^{\circ}}$C for PNNPAM [42,43,44,45], 33.7 ${}^{{}^{\circ}}$C for PNIPAM [46,47,48,49,50,51,52], 44.3 ${}^{{}^{\circ}}$C for PNIPMAM [18,19,31,53,54,55,56] and are in good agreement with literature.

Further comparison of the homopolymer swelling curves or, rather, of the phase transition width shows that PNNPAM particles exhibit a sharper transition than the other two microgels. In a temperature range of 1 ${}^{{}^{\circ}}$C (for PNNPAM between 22 °C and 23 ${}^{{}^{\circ}}$C) the hydrodynamic radius of the particles changes by about 48% with respect to the maximum volume. In the case of PNIPAM and PNIPMAM, the values are significantly smaller with 16% and 13%. Due to the fact that the PNIPMAM particles are generally larger than those made of PNNPAM and PNIPAM, the swelling ratio *α* was used for a more detailed comparison. Therefore, in Equation (10) $R_{h,\text{swollen}}$ was replaced by $R{\left(T\right)}_{h}^{3}$ which is the hydrodynamic radius at a given temperature. The calculated values for *α* as a function of temperature and the corresponding first derivatives are shown in Figure 8b,d.

The *α* *vs.* temperature curves are very similar compared to the swelling curves, and the significant difference in the phase transition width is also clearly visible in the numerical derivatives. Here, the maximum value for PNNPAM is significantly increased compared to PNIPAM and PNIPMAM. Furthermore, the temperature range in which the phase transition occurs is very small. Thus, the transition behavior of homopolymers based on NNPAM can be nearly described by a discontinuous phase transition. It should be mentioned that this sharp phase transition was also observed by Inomata *et al.* for PNNPAM macrogels [43].

The differences in the transition width can be explained by either the cross-linker distribution in the network or by the chemical structure of the used monomers. As discussed earlier (see section AFM and SLS), a pronounced cross-linker gradient in the microgel particle would result in a sharp phase transition due to the simultaneous and fast collapse of a weakly cross-linked shell. However, as confirmed by AFM and SLS, there is no significant difference between the homopolymer microgels of NNPAM and NIPAM regarding the cross-linker distribution but as confirmed by PCS these particles show a different phase transition behavior. Therefore, a strong influence of the monomer chemical structure is likely. Here, the alkyl side chain is similar for NIPAM and NIPMAM (branched isopropyl group) and differs from the linear *n*-propyl group of NNPAM. It is reported in literature that the phase behavior of linear polymers based on NIPAM [54] and NNPAM [57] also differ from each other. In short, the results show that for PNIPAM the second virial coefficient decreases continuously at the Θ-temperature (calculated from SLS measurements) in a range of 8 ${}^{{}^{\circ}}$C, while, for PNNPAM, the change is more discontinuous. Furthermore, the aggregation behavior of linear polymer chains of NIPAM and NNPAM at the Θ-temperature is different (determined by the hydrodynamic radius (PCS) and the radius of gyration (SLS)). For PNNPAM, the chain aggregation occurs directly at the Θ-temperature. Contrarily, PNIPAM shows a continuous decrease in size until a temperature of nearly 32 ${}^{{}^{\circ}}$C is reached. According to Ito *et al.* [57] and Kano *et al.* [42] different structural effects cause the sharper phase transition of PNNPAM: the *n*-propyl side chain is more flexible than the isopropyl group and a change in chain conformation at the transition temperature is more likely to occur. This flexibility of the *n*-alkyl chain additionally favors the breakup of hydrogen bonds between the solvent and the amide group of the monomer. All in all the chemical structure of the three monomers used here, play an important role with respect to the phase transition behavior of the synthesized homopolymer microgel particles.

To verify the temperature dependent phase behavior observed from the light scattering experiments, turbidity and later fluorescence measurements have also been performed and subsequently compared with the results above.

#### 3.3.2. Turbidity Measurements

For the following light attenuation measurements, a relatively slow temperature ramp of $3{\;}^{{}^{\circ}}C/h$ in combination with a fast single-measurement time (less than one second) was chosen to ensure that at any time of the measurement the microgel particles are in the equilibrium state. With this special method, it is possible to obtain, at the same time, a higher amount of data points compared to common light scattering experiments. Therefore, a precise analysis and evaluation of the phase transition of the different homopolymer microgels is possible.

**Figure 9:**
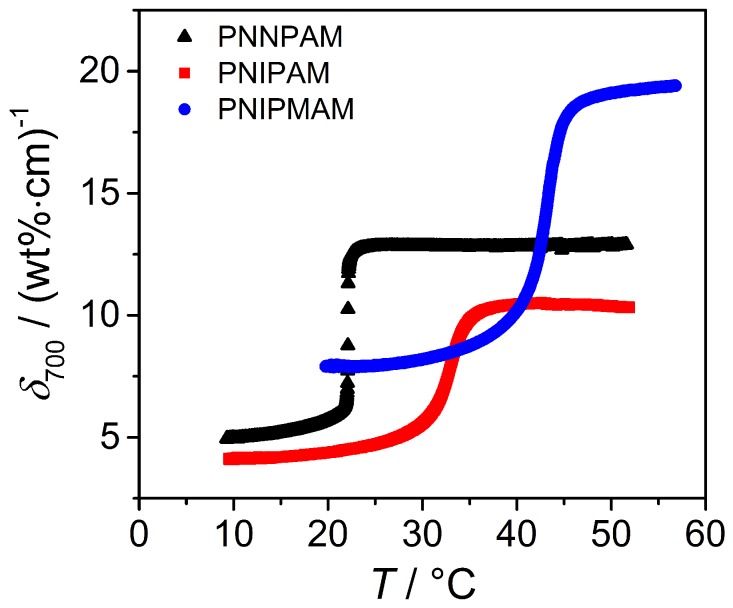
Normalized light attenuation $\delta_{700}$ at *λ* = 700 nm for PNNPAM (**black curve**), PNIPAM (**red curve**) and PNIPMAM microgels (**blue curve**) as a function of temperature measured by UV/Vis spectroscopy.

First of all, the light attenuation $D_{700}$ as a function of temperature at a wavelength of 700nm was measured. Before normalizing $D_{700}$ with respect to the microgel concentration and the thickness of the cuvette (see Equation (8)), it has to be confirmed that the light attenuation is independent of the sample concentration. Therefore, UV/Vis measurements of the homopolymer microgels at different concentrations (between 0.0015 wt % and 0.0750 wt %) were performed and a plot of $D_{700}$ as a function of concentration resulted in a line through origin which confirms the validity of the Lambert-Beer law. Hence, the normalized light attenuation $\delta_{700}$ can be calculated and is further used for a direct comparison between the different homopolymer microgels in solution. In Figure 9, the swelling curves of PNIPAM, PNNPAM and PNIPMAM homopolymers are shown.

At the VPTT, all microgel systems show an increase in the attenuation coefficient $\delta_{700}$, which is mainly caused by the change in refractive index as described in the theory section. Remarkably, the $\delta_{700}$ value at all temperatures for PNNPAM is slightly higher than for PNIPAM although the particles nearly have the same size. Therefore, the polymer based NNPAM exhibits a higher refractive index than PNIPAM. The strong deviation of PNIPMAM from the other two homopolymers is also very pronounced in the turbidity measurements, which is in line with the results from the other characterization methods. Here, the effect is mainly caused by the particle size ($R_{\text{particle}}$ from the SLS measurements: swollen state: PNNPAM/PNIPAM ≈300nm/284nm; PNIPMAM ≈464nm). Additionally, the PNNPAM microgel shows a sharper phase transition compared to PNIPAM and PNIPMAM, which was also observed in the light scattering experiments. From the high amount of data points observed by this measurement technique, a more detailed analysis of the phase transition and the determination of the VPTT is possible. The temperature dependent light attenuation coefficients are first numerically differentiated, and the numerically obtained derivative is subsequently fitted with an asymmetric Lorentz function [20,58]:

(11) $$\frac{d\delta_{\lambda }}{dT}=\frac{2a}{\pi w(T)}\cdot \frac{1}{1+4{\left[\left(T-\text{VPTT}\right)/w\left(T\right)\right]}^{2}}\;\text{with}\;w\left(T\right)=\frac{2w_{0}}{1+\exp[B(T-\text{VPTT})]}.$$

**Figure 10:**
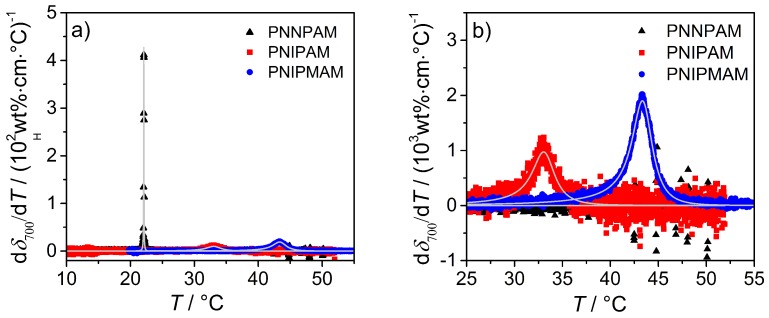
Analysis of the phase transition of PNNPAM, PNIPAM and PNIPMAM microgels using UV/Vis spectroscopy. (**a**) first derivative of the light attenuation coefficients with respect to temperature as a function of temperature; (**b**) detailed image of the temperature range between 25 ${}^{{}^{\circ}}$C and 55 ${}^{{}^{\circ}}$C relevant for PNIPAM and PNIPMAM particles.

In these equations, *a* represents the peak area, $w_{0}$ the full width at half maximum, and *B* stands for the asymmetry of the function. At a value of zero for *B*, a symmetric Lorentz function can be obtained. The results from the analysis of the first derivative of the light attenuation curves and the corresponding asymmetric Lorentz fits are shown in Figure 10. To provide a clearer presentation of the PNIPAM and PNNPAM data, the curves are enlarged in the relevant temperature region (see Figure 10b). The obtained values of the transition temperature and the full width at half maximum $w_{0}$ are listed in Table 4.

The VPTT of the homopolymer microgels are in good agreement with the values from the light scattering experiments. It is notable that the microgel based on NNPAM generally exhibits a steeper phase transition compared to the other two polymers. This fact is clearly visible in the UV/Vis spectra and also in the PCS measurements.

**Figure 11:**
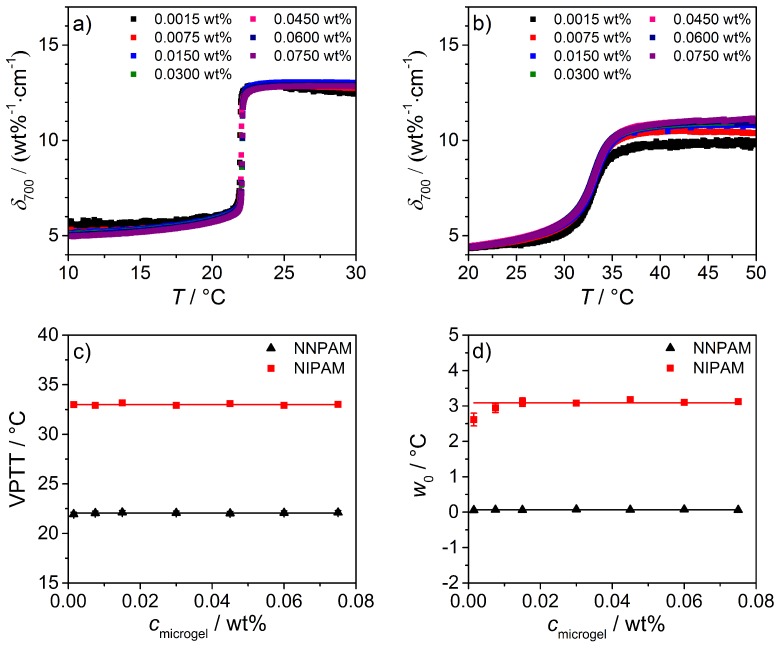
Normalized light attenuation coefficients for PNNPAM (**a**) and PNIPAM (**b**) microgels solutions with different concentrations as a function of temperature (*λ* = 700 nm). In graphs (**c**) and (**d**), a summary of the VPTTs and the $w_{0}$-values for both microgels as a function of sample concentration is given. The solid lines correspond to the average value. It has to be mentioned that for PNIPAM the first $w_{0}$-value (0.0015 wt %) has been neglected.

Furthermore, the influence of the microgel concentration on the characteristics of the phase transition ($w_{0}$ the full width at half maximum and VPTT) has been analyzed. For this purpose, the temperature dependent $\delta_{700}$ values of the homopolymer microgels at different concentrations have been plotted as a function of temperature, and the results for PNNPAM and PNIPAM are depicted in Figure 11. From the graphs, it is obvious that the normalized swelling curves of the microgels with varying concentration show a similar behavior, and, consequently, the phase transition of the homopolymer microgels is independent of the sample concentration (see Figure 11a,b).

Afterwards, the curves of the phase transition have been numerically differentiated and fitted by the asymmetric Lorentz function from Equation (11). The calculated values for the VPTT and $w_{0}$ as a function of the microgel concentration are summarized in Figure 11c,d. It is clearly visible that both parameters are independent of the concentration. Only for PNIPAM at the lowest concentration of 0.0015 wt %, a slight deviation of $w_{0}$, is recognizable. This is due the low signal during the phase transition and the corresponding low change in the $\delta_{700}$ value. Therefore, the respective data point in the plot of $w_{0}$ *vs.* $c_{\text{microgel}}$ was neglected for the estimation of the average value.

The presented concentration dependent measurements show that the light attenuation and PCS experiments with respect to the phase transition behavior, the VPTT, and $w_{0}$ are in good agreement. Therefore, the attenuation measurement is well suited for the evaluation of the phase behavior of different microgel solutions and is a good alternative to PCS.

#### 3.3.3. Fluorescence Measurements

Until now, the phase transition behavior of the homopolymer microgels has been characterized by PCS experiments (changes in the hydrodynamic properties) and by turbidity measurements (changes in the scattering ability). But these two methods provide no information on the processes occurring on a molecular level inside the microgel particle. One possibility to obtain knowledge about the interior of the colloidal gel particles is to investigate polarity changes during the phase transition. For this purpose, the homopolymers have been dispersed in a saturated solution of pyrene and the phase behavior was followed by fluorescence spectroscopy. To our knowledge, this technique was net yet applied to study the volume phase transition of thermoresponsive microgels. However, the swelling behavior of a NIPAM-dye copolymer system, where the dye molecule is covalently bound to a linear PNIPAM chain, was investigated by Matsumura [59].

After illumination of a pure pyrene solution with light of a wavelength of 355nm, four characteristic fluorescence bands can be found. Here, the intensity ratio of $I_{1}$ at an emission wavelength of 372nm to $I_{3}$ at 382nm depends on the polarity of the environment. According to Kalyanasudaram *et al.* [60], the absolute value $I_{1}/I_{3}$ can therefore serve as a direct measure of the environment polarity. Generally, if the value $I_{1}/I_{3}$ is high, the polarity is high and the environment is more hydrophilic. However, the work of Kalyanasudaram is based on different pure solvents with varying polarities where only the solvent molecules define the environment polarity. In the case of a microgel solution, not only the solvent contributes to the overall polarity of the system but also the polymer. Here, part of the dissolved pyrene molecules can be outside of the microgel particles (surrounded by pure solvent) and another fraction of the pyrene is localized inside the gel network and the hydrophilic/hydrophobic character of the polymer chains also contributes. Therefore, the measured intensity ratio $I_{1}/I_{3}$ will be always an average of both contributions. In responsive microgels based on acrylamides, the hydrophobic properties increase during the phase transition and so pyrene and the intensity ratio $I_{1}/I_{3}$ of the fluorescence bands can be used as a sensor to follow the local polarity inside the particles during the phase transition [33,61,62,63].

**Figure 12:**
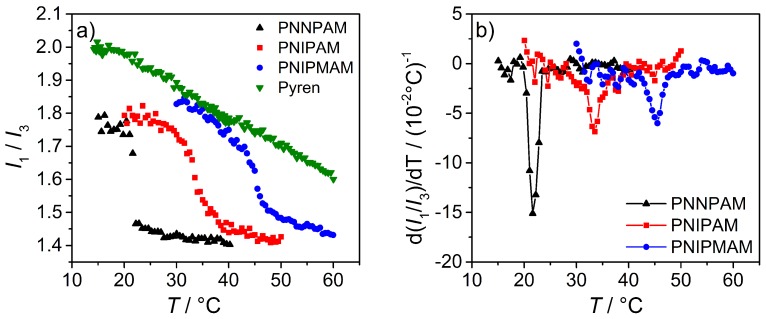
(**a**) Plot of the intensity ratio $I_{1}/I_{3}$ as a function of temperature for PNNPAM, PNIPAM and NIPMAM solutions mixed with pyrene as well as pure pyrene as reference; (**b**) To quantify the VPTT, the first derivative of $I_{1}/I_{3}$ with respect to temperature as a function of temperature is presented. The concentration of all microgel solutions was 0.1 wt %.

For the fluorescence experiments, a microgel concentration of 0.1 wt % in a saturated pyrene solution was used and the $I_{1}/I_{3}$ ratio as a function of temperature was measured. To ensure that the polymer system is in equilibrium at any point, an additional waiting time of 10 min after every temperature step was chosen. The results of the temperature dependent intensity ratios for the homopolymers of PNNPAM, PNIPAM, PNIPMAM and the reference system pyrene are shown in Figure 12a). Additionally, on the right hand side of Figure 12, the corresponding first derivative of the swelling curves are given.

The fluorescence based swelling curves of all three homopolymers clearly show a decrease in polarity with an increase in temperature where the effect is most pronounced at the VPTT. In this temperature region, the polymer network collapses and changes its hydrophobicity. At the same time, one part of the pyrene molecules is caged inside the gel network and the other fraction is of course expelled into the solvent. The measured decrease in $I_{1}/I_{3}$ is not an absolute value for the polarity of the particle interior (as described before), but the trend of the fluorescence swelling curves is in good agreement with the results from the PCS and turbidity measurements. This shows the feasibility of the study of the swelling behavior by the presented fluorescence technique. PNNPAM again shows the steepest phase transition compared to PNIPAM and PNIPMAM, which is related to a dramatic change from a hydrophilic to a more hydrophobic structure connected with a strong decrease in particle size.

A closer look at the phase transition curves in Figure 12 shows an additional slight decrease in the intensity ratio before and after the VPTT. This change in $I_{1}/I_{3}$ with increasing temperature is also observable for the pure pyrene solution. The reason for this is a reduction of the dielectric constant of the solvent as a function of temperature and as a consequence, the interactions between pyrene and the water molecules are reduced [64]. Hence, the slight linear decrease in the intensity ratio before and after the VPTT is not caused by the collapse of the microgel particles, but by the interactions of pyrene with the solvent.

The absolute values for $I_{1}/I_{3}$ in the swollen as well as in the collapsed state of the microgels decrease from PNIPMAM over PNIPAM to PNNPAM. This suggests that the hydrophilic character of the polymers is more pronounced for PNIPMAM and the lowest for PNNPAM. This trend is consistent with the location of the VPTTs, and here the transition temperature is higher for more hydrophilic polymers. Winnik *et al.* [65] observed a similar behavior for linear PNIPAM copolymerized with different hydrophobic *N-n*-alkyl acrylamides. The absolute value for $I_{1}/I_{3}$ decreased with an increasing content of the hydrophobic comonomer.

**Figure 13:**
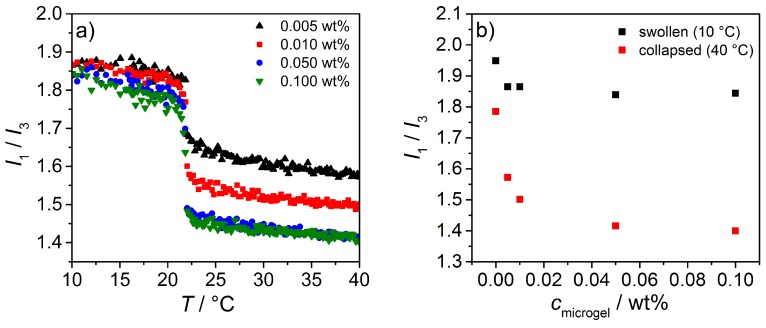
Plot of the intensity ratio $I_{1}/I_{3}$ as a function of temperature for PNNPAM microgel solutions with different concentrations (**left**). Additionally, the concentration dependent change in $I_{1}/I_{3}$ for the swollen and collapsed microgel is shown (**right**).

To check if there is a saturation concentration of the microgel particles interacting with pyrene, microgel concentration dependent fluorescence measurements were performed to determine the change in $I_{1}/I_{3}$. The results are summarized in Figure 13. The left graph shows that, for the microgel particles in the collapsed state, the intensity ratio decreases continuously with increasing microgel concentration until a certain threshold value is reached. This can be attributed to the effect that at higher microgel concentrations more particles are available to interact with the pyrene molecules. As soon as a concentration of 0.050wt% is reached, the intensity ratio shows the similar behavior. Accordingly, measurements performed at these concentrations, can be easily compared. In contrast to this the microgel/pyrene mixtures show at low temperatures nearly no temperature dependent behavior.

Based on all results shown in this section, it is clear that the hydrophilicity of the microgel particles increases from monomer NNPAM to NIPAM to NIPMAM.

## 4. Conclusions

We have studied the influence of the monomer structure on the properties of the respective synthesized microgels employing the monomers NNPAM, NIPAM and NIPMAM. The used experimental conditions were identical for all three microgel types. PNNPAM microgels are found to stand out since they exhibit a very sharp VPT. However, concerning particle size and cross-linker gradient, PNNPAM and PNIPAM microgels are very similar. Both are found to exhibit a core-shell structure with a harder, strongly cross-linked core and a fuzzy shell. PNIPMAM particles are much more homogeneous, which was revealed in the AFM phase images. This points to a different formation mechanism of the PNIPMAM microgels. Moreover, we have shown that fluorescence measurements with pyrene as a probe can be applied to follow the volume phase transition of smart microgels.

## Figures and Tables

**Table 1:** Particle diameters obtained by AFM and SEM. Additionally, the relationship of the diameters to each other was calculated.

Microgel	${\bar{D}}_{\text{SEM}}$/nm	${\bar{D}}_{\text{AFM}}$/nm	${\bar{D}}_{\text{SEM}}/{\bar{D}}_{\text{AFM}}$
NNPAM	467 ± 83	790 ± 71	0.59
NIPAM	461 ± 90	776 ± 79	0.59
NIPMAM	769 ± 90	786 ± 89	0.98

**Table 2:** Results from the fit of the experimental static light scattering (SLS) data from the homopolymer microgels with the fuzzy sphere form factor model.

Microgel	$q_{\text{min}}/\left(10^{-2}{nm}^{-2}\right)$	$R_{\text{particle}}$/nm	${PD}_{\text{particle}}$/%	*σ*/nm
NNPAM	1.73	300	5.2	20
NIPAM	1.77	284	6.8	16
NIPMAM	1.35	464	5.6	67

**Table 3:** Summary of the hydrodynamic radii of the homopolymer microgels in the swollen and collapsed state in combination with the maximum swelling ratio $\alpha_{\text{max}}$. The error of the radii corresponds to an estimated deviation of 5%. This value includes not only the inaccuracy of the measurement but also the very low polydispersity of the sample.

Microgel	$R_{h,\text{swollen}}$/nm	$R_{h,\text{collapsed}}$/nm	$\alpha_{\text{max}}$
NNPAM	291±15	146±7	7.9±1.7
NIPAM	297±15	139±7	9.8±2.1
NIPMAM	431±22	211±11	8.5±1.8

**Table 4:** Volume phase transition temperatures of the homopolymer microgels obtained by turbidity measurements. $w_{0}$ is the full width at half maximum of the phase transition peak from the Lorentz approximation.

Microgel	VPTT/${}^{{}^{\circ}}$C	$w_{0}$/${}^{{}^{\circ}}$C
NNPAM	22.1 ± 0.1	0.06 ± 0.01
NIPAM	33.0 ± 0.1	3.12 ± 0.01
NIPMAM	43.2 ± 0.1	2.70 ± 0.01
